# Mortality in Yusho patients exposed to polychlorinated biphenyls and polychlorinated dibenzofurans: a 50-year retrospective cohort study

**DOI:** 10.1186/s12940-020-00680-0

**Published:** 2020-11-23

**Authors:** Daisuke Onozuka, Yuko Nakamura, Gaku Tsuji, Masutaka Furue

**Affiliations:** 1 Department of Preventive Medicine and Epidemiology, National Cerebral and Cardiovascular Center Research Institute, 6-1 Kishibeshin-machi, Suita, Osaka, 564-8565 Japan; 2 Department of Health Care Administration and Management, Graduate School of Medical Sciences, Kyushu University, Fukuoka, Japan; 3 Research and Clinical Center for Yusho and Dioxin, Kyushu University Hospital, Fukuoka, Japan; 4 Department of Dermatology, Graduate School of Medical Sciences, Kyushu University, Fukuoka, Japan

**Keywords:** Polychlorinated biphenyls, Polychlorinated dibenzofurans, Cancer, Cohort study, Yusho

## Abstract

**Background:**

In 1968, the Yusho incident resulted in accidental exposure to polychlorinated biphenyls (PCBs), polychlorinated dibenzofurans (PCDFs), and related compounds in Japan. This study updated the risk of mortality in Yusho patients.

**Methods:**

We obtained updated cohort data for all Yusho patients for the period 1968–2017. We calculated standardized mortality ratios (SMRs) for all-cause and cause-specific mortality over a 50-year follow-up period compared with the general population in Japan.

**Results:**

A total of 1664 Yusho patients with 63,566 person-years of follow up were included in the analysis. Among males, excess mortality was observed for all cancers (SMR: 1.22, 95% confidence interval [CI]: 1.02 to 1.45) and lung cancer (SMR: 1.59, 95% CI: 1.12 to 2.19). Among females, increased mortality was observed for liver cancer (SMR: 2.05, 95% CI: 1.02 to 3.67). No significant increase was seen in non-cancer-related mortality compared with the general population.

**Conclusions:**

Carcinogenic risk in humans after exposure to PCBs and PCDFs remains higher among Yusho patients. Our findings suggest the importance of care engagement and optimum management to deal with the burden of Yusho disease.

## Introduction

In 1968, a mass food poisoning incident involving more than 1800 patients occurred in western Japan [1]. The incident was called Yusho (oil disease in Japanese) since it was caused by the ingestion of rice bran oil which had been contaminated with polychlorinated biphenyls (PCBs), polychlorinated dibenzofurans (PCDFs), and other dioxin-related compounds [2, 3]. This accidental release of PCBs and PCDFs into rice bran oil led to acute and chronic toxicity, and Yusho patients have suffered a broad range of symptoms such as comedones, acneiform eruption, and pigmentation of the conjunctiva, skin, and gingiva [4, 5]. Following the Yusho incident, a comparable mass food poisoning incident occurred in central Taiwan in 1979, Yucheng (oil disease in Chinese), which was also caused by the ingestion of cooking oil contaminated with PCBs and dioxins [6]. There is a large variation in half-lives between different PCB congeners depending on the number and position of the chlorine atoms; highly chlorinated congeners tend to remain in the body longer than do less-chlorinated congeners [7–9]. Dioxins generally show lipophilic and biologically stability in the body [10], remain in adipose tissue for an extended period, and can lead to the development of long-term symptoms [10]. The Yusho and Yucheng incidents have therefore been used to evaluate the long-term adverse health effects associated with the ingestion of PCBs and PCDFs.

Our 40-year follow-up study of the Yusho cohort revealed elevated mortality from all cancers, lung cancer, and liver cancer in males [11]. A meta-analysis of the Yusho and Yucheng cohorts also showed increased mortality from all cancers, lung cancer, heart conditions, and liver conditions among males and liver cancer among females [12]. Further longer-term investigation to determine whether cancer risk remains high may increase our understanding of the disease, but no 50-year follow-up evaluation of Yusho patients has yet been reported.

Here, we report data on a 50-year follow-up evaluation of Yusho patients and updated the risk of mortality.

## Methods

### Data sources

**Fig. 1:**
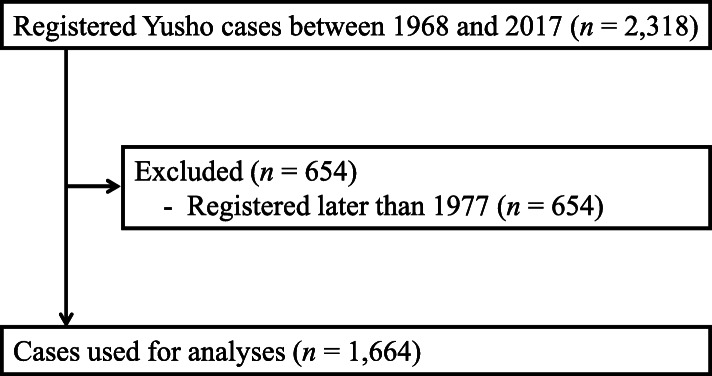
Yusho data included in the study

The Yusho registry was begun in 1968 and Yusho patients for this report were followed up until December 2017 or death. For patients who became lost to follow-up, vital status data was collected on the last date on which they were known to be alive. As of December 31, 2017, a total of 2318 Yusho patients were registered in the Yusho case registry. In Japan, registration of Yusho data is required under the Act on the Comprehensive Promotion of Policy for Yusho Patients and is considered to have a same validity and completeness between prefectures and different years. We excluded 654 subjects who were registered later than 1977 and had not been diagnosed as having Yusho from the outset of the Yusho incident because the diagnostic criteria for Yusho were revised in several times after the Yusho incident and these factors could lead to miss potentially affected patients who died prior to the official registration period and potentially introduce bias [11]. After exclusion of these cases, a total of 1664 subjects met the inclusion criteria (Fig. 1). None of the subjects joined a specific medical surveillance program to early detect PCB/PCDF-related diseases or received a special treatment.

The subjects were followed retrospectively from 1968 to 2017. Details of the follow-up survey used for Yusho patients have been previously presented [11, 13, 14]. In brief, all subjects were identified by name, sex, age, date of birth, home address, as well as date and place of registration. To determine the vital status of Yusho patients, follow-up surveys were conducted under the cooperation of the Ministry of Health, Labour and Welfare and prefectural and municipal government. In Japan, residency and death registration are legally required, and the registries are considered to be complete. Data on underlying causes of death are reported electronically from the municipal government to the Ministry of Health, Labour and Welfare. Death certificates are required to be completed by a licensed physician and are considered reliable with regard to both quality and completeness.

We linked data on the Yusho cohort from 1968 to 2017 to the Ministry of Health, Labour and Welfare national mortality register using record linkage, which has been confirmed reliable and valid in tracing targeted individuals [15]. Actual causes of deaths were described based on the International Classification of Diseases. On successful linkage, we extracted the *International Classification of Diseases* code, and then adapted codes to those of the Ninth Revision to ensure consistency with the underlying cause data, standardization, and compatibility of mortality rates.

### Statistical analysis

Person-years at risk were calculated beginning at the official registration date for each Yusho patient until the time of death or end of follow-up, whichever was first recorded. We selected the official registration date as the start date in obtaining person-years due to difficulties in identifying target individuals at first exposure; the contaminated oil was broadly distributed in western Japan, and it was not possible to determine the amount of exposure to PCBs and PCDFs at the time the Yusho incident occurred. Thus, some potential person-years between the date of exposure and the date of registration may have been missed. For patients who were unavailable for follow-up, person-years were censored on the last date on which they were known to be alive. Person-years were stratified by gender, 10-year age groups, and 10-year calendar periods. The expected number of deaths due to all causes and cause-specific diseases was calculated using life-table methods, with the application of reference mortality rates for the general Japanese population by attained age and calendar period. The sex-specific national death rates for 10-year age and 10-year calendar-period intervals were used as reference rates.

To compare the Yusho cohort mortality to the general Japanese population, we calculated standardized mortality ratios (SMRs) for each outcome by dividing the number of deaths observed by that of deaths expected. We calculated 95% confidence intervals (CIs) for the SMRs, assuming that the occurrence of events followed a Poisson distribution. All causes and cause-specific SMRs were calculated according to sex and 10-year calendar-period intervals since the Yusho accident.

All statistical analyses were performed using Stata 16.1 (StataCorp LLC, College Station, TX, USA). We used a 2-sided *P* value of 0.05 to define statistical significance.

## Results

Table 1 shows the characteristics of the Yusho cohort. A total of 1664 Yusho patients were included in the analysis (Fig. 1), of whom 643 (38.6%) had died during the follow-up period. Total follow-up time was 63,566 person years (31,559 person years for males and 32,007 person years for females) and median follow-up time was 43.7 years (42.9 years for males and 44.6 years for females). The mean age of Yusho patients confirmed alive in 2017 was 66.3 years (64.9 years for males and 67.6 years for females). Vital status and cause of death were determined for 1584 Yusho patients (95.2%).

**Table 1:** Distribution by age in 1968 versus vital status in 2017 of Japanese Yusho patients

	Males (*n* = 860)	Females (*n* = 804)
	No. (%)	No. (%)
Age in 1968		
< 20	345 (40.1)	305 (37.9)
20–29	94 (10.9)	99 (12.3)
30–39	126 (14.7)	136 (16.9)
40–49	140 (16.3)	130 (16.2)
50–59	82 (9.5)	68 (8.5)
60–69	49 (5.7)	43 (5.3)
70–79	20 (2.3)	21 (2.6)
> 80	4 (0.5)	2 (0.2)
Vital status of 31 December 2017		
Alive	500 (58.1)	521 (64.8)
Dead	360 (41.9)	283 (35.2)
Person-years of follow-up	31,559	32,007

Tables 2 shows the number of deaths both observed and expected, SMRs, and 95% CIs of the major causes of death in Japanese Yusho patients between 1968 and 2017. During the 50-year follow-up period, all-cause mortality in the Yusho cohort was comparable to that in the Japanese general population (SMR for males: 1.04, 95% CI: 0.94 to 1.15; SMR for females: 1.05, 95% CI: 0.93 to 1.17). Among males, excess mortality was observed for all cancers (SMR: 1.22, 95% CI: 1.02 to 1.45) and lung cancer (SMR: 1.59, 95% CI: 1.12 to 2.19), and there was higher mortality from liver cancer (SMR: 1.49, 95% CI: 0.90 to 2.33). Among females, increased mortality was observed for liver cancer (SMR: 2.05, 95% CI: 1.02 to 3.67). No significant increase was found in non-cancer-related mortality on comparison with the general population.

**Table 2:** Numbers of observed and expected deaths, SMRs, and 95% CI for the major causes of death in Japanese Yusho patients, 1968 and 2017

Cause of death (ICD–9 codes)	Males					Females				
	Observed, no.	Expected, no.	SMR	95% CI		Observed, no.	Expected, no.	SMR	95% CI	
All causes (001–999)	360	346.2	1.04	0.94,	1.15	283	270.8	1.05	0.93,	1.17
All cancers (140–208)	132	108.3	1.22	1.02,	1.45	66	67.9	0.97	0.75,	1.24
Stomach (151)	24	22.3	1.07	0.69,	1.60	5	11.8	0.43	0.14,	0.99
Rectum (154)	4	4.5	0.88	0.24,	2.25	2	2.7	0.75	0.08,	2.70
Liver (155)	19	12.8	1.49	0.90,	2.33	11	5.4	2.05	1.02,	3.67
Pancreas (157)	8	6.5	1.24	0.53,	2.44	7	5.2	1.34	0.54,	2.75
Lung (162)	37	23.3	1.59	1.12,	2.19	8	8.1	0.99	0.42,	1.94
Breast (174)	0	0.0				6	5.2	1.15	0.42,	2.51
Uterus (179–182)						5	3.6	1.39	0.45,	3.25
Leukemia (204–208)	2	2.5	0.81	0.09,	2.93	0	1.7	0.00	0.00,	0.00
Diabetes mellitus (250)	1	4.1	0.24	0.00,	1.36	3	3.7	0.82	0.16,	2.38
Hypertension (401–405)	2	2.6	0.77	0.09,	2.77	1	3.4	0.29	0.00,	1.62
Heart disease (393–398, 410–429)	54	51.3	1.05	0.79,	1.37	51	47.8	1.07	0.79,	1.40
Cerebrovascular disease (430–438)	39	46.2	0.84	0.60,	1.15	44	42.7	1.03	0.75,	1.38
Hepatic disease (570–573)	12	8.5	1.42	0.73,	2.49	4	3.7	1.07	0.29,	2.73

Abbreviation: *ICD–9* International Classification of Diseases, Ninth Revision, *SMR* Standardized mortality ratio, *CI* Confidence interval

Tables 3 and 4 show the observed and expected number of deaths, SMRs, and 95% CIs for the major causes of death among male and female Japanese Yusho patients by 10-year intervals from 1968 to 2017. Among males, excess mortality was observed for all cancers during the first and second 10-year period (SMR for 1968–1977: 2.48, 95% CI: 1.42 to 4.03; SMR for 1978–1987: 1.69, 95% CI: 1.11 to 2.46). However, such increases in mortality were not found after the third 10-year period. For females, there was no significant difference in mortality for the major causes of death for any calendar period.

**Table 3:** Numbers of observed and expected deaths, SMRs, and 95% CI for major causes of death in male Japanese Yusho patients in 10-year intervals, 1968 to 2017

Cause of death (ICD–9 codes)	Number of years since the accident																								
	0–9 (1968–1977)					10–19 (1978–1987)					20–29 (1988–1997)					30–39 (1998–2007)					40–49 (2008–2017)				
	Obs, no.	Exp, no.	SMR	95% CI		Obs, no.	Exp, no.	SMR	95% CI		Obs, no.	Exp, no.	SMR	95% CI		Obs, no.	Exp, no.	SMR	95% CI		Obs, no.	Exp, no.	SMR	95% CI	
All causes (001–999)	43	32.3	1.33	0.96,	1.79	73	58.8	1.24	0.97,	1.56	72	70.4	1.02	0.80,	1.29	86	86.7	0.99	0.79,	1.23	86	99.9	0.86	0.69,	1.06
All cancers (140–208)	16	6.4	2.48	1.42,	4.03	27	16.0	1.69	1.11,	2.46	25	23.2	1.08	0.70,	1.59	37	30.0	1.23	0.87,	1.70	27	32.5	0.83	0.55,	1.21
Stomach (151)	9	2.7	3.35	1.53,	6.35	3	4.8	0.62	0.13,	1.82	5	5.0	1.01	0.33,	2.35	4	5.2	0.76	0.21,	1.95	3	4.8	0.62	0.13,	1.82
Rectum (154)	0	0.2	0.00	0.00,	14.80	0	0.7	0.00	0.00,	5.60	2	1.0	2.03	0.25,	7.32	0	1.3	0.00	0.00,	2.90	2	1.4	1.46	0.18,	5.27
Liver (155)	3	0.6	5.18	1.07,	15.15	5	2.0	2.55	0.83,	5.96	6	3.4	1.78	0.65,	3.87	3	3.7	0.81	0.17,	2.37	2	3.0	0.66	0.08,	2.38
Pancreas (157)	0	0.3	0.00	0.00,	14.17	2	0.8	2.41	0.29,	8.69	1	1.3	0.76	0.02,	4.24	3	1.8	1.68	0.35,	4.91	2	2.3	0.88	0.11,	3.18
Lung (162)	2	0.8	2.55	0.31,	9.21	9	2.8	3.23	1.48,	6.14	5	4.9	1.03	0.33,	2.39	12	6.9	1.74	0.90,	3.03	9	7.9	1.14	0.52,	2.17
Breast (174)	0	0.0				0	0.0				0	0.0				0	0.0				0	0.0			
Uterus (179–182)																									
Leukemia (204–208)	0	0.2	0.00	0.00,	17.05	2	0.4	4.94	0.60,	17.85	0	0.5	0.00	0.00,	7.37	0	0.6	0.00	0.00,	5.96	0	0.7	0.00	0.00,	5.17
Diabetes mellitus (250)	1	0.4	2.85	0.07,	15.90	0	0.6	0.00	0.00,	5.74	0	0.9	0.00	0.00,	4.13	0	1.1	0.00	0.00,	3.43	0	1.1	0.00	0.00,	3.28
Hypertension (401–405)	0	0.7	0.00	0.00,	5.60	1	0.8	1.22	0.03,	6.82	0	0.5	0.00	0.00,	8.08	1	0.3	2.95	0.07,	16.45	0	0.4	0.00	0.00,	9.13
Heart disease (393–398, 410–429)	7	4.0	1.76	0.71,	3.63	10	9.9	1.01	0.48,	1.85	10	11.4	0.88	0.42,	1.62	11	12.3	0.90	0.45,	1.61	16	14.2	1.13	0.65,	1.84
Cerebrovascular disease (430–438)	7	7.6	0.92	0.37,	1.89	10	10.8	0.92	0.44,	1.70	9	9.3	0.97	0.44,	1.84	8	10.2	0.78	0.34,	1.54	5	9.1	0.55	0.18,	1.28
Hepatic disease (570–573)	2	1.1	1.74	0.21,	6.30	4	2.2	1.80	0.49,	4.60	4	2.0	2.05	0.56,	5.25	1	1.6	0.61	0.02,	3.40	1	1.5	0.66	0.02,	3.69

Abbreviation: *Exp* Expected, *ICD–9* International Classification of Diseases, Ninth Revision, *Obs* Observed, *SMR* Standardized mortality ratio, *CI* Confidence interval

**Table 4:** Observed and expected numbers of deaths, SMRs, and 95% CI for major causes of death in female Japanese Yusho patients in 10-year intervals, 1968 to 2017

Cause of death (ICD–9 codes)	Number of years since the accident																								
	0–9 (1968–1977)					10–19 (1978–1987)					20–29 (1988–1997)					30–39 (1998–2007)					40–49 (2008–2017)				
	Obs, no.	Exp, no.	SMR	95% CI		Obs, no.	Exp, no.	SMR	95% CI		Obs, no.	Exp, no.	SMR	95% CI		Obs, no.	Exp, no.	SMR	95% CI		Obs, no.	Exp, no.	SMR	95% CI	
All causes (001–999)	19	24.2	0.79	0.47,	1.23	37	41.4	0.89	0.63,	1.23	61	51.1	1.19	0.91,	1.53	65	65.2	1.00	0.77,	1.27	101	91.3	1.11	0.90,	1.34
All cancers (140–208)	4	4.8	0.83	0.23,	2.12	4	10.0	0.40	0.11,	1.03	12	13.6	0.88	0.46,	1.54	17	18.2	0.94	0.54,	1.50	29	21.4	1.35	0.91,	1.94
Stomach (151)	0	1.7	0.00	0.00,	2.24	0	2.6	0.00	0.00,	1.41	2	2.5	0.79	0.10,	2.85	1	2.6	0.38	0.01,	2.13	2	2.5	0.81	0.10,	2.93
Rectum (154)	0	0.2	0.00	0.00,	18.13	0	0.4	0.00	0.00,	8.79	0	0.6	0.00	0.00,	6.64	1	0.7	1.42	0.04,	7.89	1	0.8	1.27	0.03,	7.06
Liver (155)	1	0.3	3.38	0.09,	18.85	1	0.6	1.57	0.04,	8.77	1	1.2	0.87	0.02,	4.84	4	1.7	2.37	0.65,	6.06	4	1.6	2.53	0.69,	6.48
Pancreas (157)	0	0.2	0.00	0.00,	20.73	0	0.5	0.00	0.00,	6.97	2	0.9	2.14	0.26,	7.71	2	1.5	1.37	0.17,	4.95	3	2.1	1.41	0.29,	4.12
Lung (162)	0	0.3	0.00	0.00,	12.81	1	0.9	1.10	0.03,	6.10	2	1.6	1.28	0.15,	4.61	1	2.4	0.43	0.01,	2.37	4	3.0	1.34	0.37,	3.43
Breast (174)	1	0.3	3.54	0.09,	19.71	0	0.7	0.00	0.00,	5.38	1	1.0	1.00	0.03,	5.58	1	1.4	0.71	0.02,	3.93	3	1.8	1.67	0.34,	4.87
Uterus (179–182)	1	0.6	1.78	0.05,	9.91	0	0.7	0.00	0.00,	4.92	2	0.7	2.88	0.35,	10.41	0	0.8	0.00	0.00,	4.77	2	0.9	2.34	0.28,	8.46
Leukemia (204–208)	0	0.2	0.00	0.00,	22.90	0	0.3	0.00	0.00,	13.41	0	0.3	0.00	0.00,	11.02	0	0.4	0.00	0.00,	9.00	0	0.5	0.00	0.00,	7.90
Diabetes mellitus (250)	0	0.3	0.00	0.00,	10.95	0	0.6	0.00	0.00,	6.08	1	0.8	1.22	0.03,	6.78	1	0.9	1.10	0.03,	6.13	1	1.0	0.99	0.03,	5.53
Hypertension (401–405)	0	0.7	0.00	0.00,	5.00	0	1.0	0.00	0.00,	3.82	1	0.6	1.54	0.04,	8.60	0	0.5	0.00	0.00,	7.30	0	0.7	0.00	0.00,	5.42
Heart disease (393–398, 410–429)	1	3.4	0.30	0.01,	1.66	10	7.6	1.31	0.63,	2.41	18	9.7	1.85	1.10,	2.92	8	11.3	0.71	0.30,	1.39	14	16.2	0.86	0.47,	1.45
Cerebrovascular disease (430–438)	3	6.2	0.48	0.10,	1.41	8	9.2	0.87	0.38,	1.72	10	8.8	1.13	0.54,	2.09	14	9.7	1.44	0.79,	2.41	9	9.7	0.93	0.43,	1.77
Hepatic disease (570–573)	0	0.5	0.00	0.00,	7.55	4	0.8	4.91	1.34,	12.56	0	0.9	0.00	0.00,	4.23	0	0.8	0.00	0.00,	4.69	0	0.8	0.00	0.00,	4.48

Abbreviation: *Exp* Expected, *ICD–9* International Classification of Diseases, Ninth Revision, *Obs* Observed, *SMR* Standardized mortality ratio, *CI* Confidence interval

## Discussion

Here, we provide a 50-year follow-up of Japanese Yusho patients. Our data showed that male Yusho patients had a significant increased risk of mortality from all cancer and lung cancer. Compared with our previous study, the new finding is that significant excess mortality in liver cancer was observed among female Yusho patients in the 50-year follow-up. Our findings suggest that carcinogenic risk to humans after exposure to PCBs and PCDFs remains higher among Yusho patients.

Previous results regarding the associations between PCB and dioxin exposure and mortality have been inconsistent. After 40 years of follow-up, we observed no significant difference in death due to all-cause for either male or female Yusho patients when compared with the Japanese general population [11]. We also estimated that the relative survival and net survival of Yusho patients were around those expected from national rates [13, 14]. In contrast, there was significant increase in all-cause mortality for male Yucheng patients (as noted, “Yucheng” means “oil disease” in Chinese) on follow up for 30 years among Taiwanese people with high exposure to PCBs and PCDFs [16]. Additionally, a meta-analysis of Yusho and Yucheng cohorts found increased all-cause mortality in males [12]. Apart from these Yusho and Yucheng studies, a ten-year follow-up study of employees exposed to PCBs in three electrical capacitor manufacturing factories suggested that all-cause mortality was not increased [17]. Further, a long-term follow-up study of capacitor workers exposed to PCBs suggested that mortality for all causes was significantly lower in the total cohort [18]. These discrepancies were possibly ascribable to confounding and use of mortality in the general population, follow-up period, and exposure levels between studies. Especially in our study, national mortality rates for major causes of death were used as reference points and the follow-up period was stated from the date of registration. Moreover, in our study, the definition of exposure was certified patients in the registry.

Fifty years after the Yusho incident, there was a statistically significant elevation in the risk of all-cancer mortality in male Yusho patients. These results conform with those of a previous Yusho cohort [11] and a meta-analysis of the Yusho and Yucheng patients [12]. Further, we observed increased cancer mortality in the early years after exposure to PCBs and PCDFs, and there are no significantly increased SMRs for cancer in the last 30 years of the follow-up period. This might be considered that chemical carcinogens cause cancer, but with certain latency period. Among people who were exposed to human carcinogens, it is generally believed that certain induction time or latency period is required before excess cancer can be observed. Patients are asymptomatic or inapparent during the induction time or latency period. After the disease process has been triggered, pathophysiological changes occur without the individual being aware of them and the adverse effects associated with cancer may be accelerated. Thus, if the excess of cancer mortality was excluded during this period, the increased SMRs might not be observed in Yusho patients. Our results suggest the importance to consider latency period for the development of cancer after chemical exposure [19, 20]. In 2013, based on sufficient proof for carcinogenicity in both humans and animals, a Working Group of the International Agency for Research on Cancer (IARC) determined that there was sufficient evidence of carcinogenicity for PCBs in humans, and classified PCBs as carcinogenic in humans (Group 1) [21]. However, data for site-specific cancers were insufficient to draw conclusions. In 2015, although the working group published their findings in detail and showed the data, most of the secured data referred only to the malignant melanoma [22]. Further investigation on holding combination studies with the Yucheng cohort is warranted.

Our study of Yusho patients with 50-year follow-up found that there was significant increased mortality for liver cancer among females. For males, mortality for liver cancer and hepatic disease elevated, but not significantly so. These results are consistent with our study with 40-year follow-up on the Yusho cohort [11]. In contrast, a similar 30-year follow-up study of the Yucheng cohort found elevated mortality from liver disease, whereas no increase in the risk of cancer of the liver [16]. The excess mortality due to all cancers and liver cancer found in Yusho patients was not observed in Yucheng patients. These results might be ascribable to differences in the composition of PCDF isomers between the Yusho and Yucheng incident: the main PCDF isomer in Yucheng patients was 1,2,3,4,7,8-hexachlorinated dibenzofuran, whereas that in Yusho patients was 2,3,4,7,8-pentachlorinated dibenzofuran, which has a greater toxic equivalency factor than that in the Yucheng incident [11]. Additionally, the differences may be due to diagnostic misclassification of liver cancer and other liver diseases [23]. The GLOBOCAN estimates has shown hepatocellular carcinoma (HCC) to be the most prevalent primary liver cancer [24], which accounts for 91.9% of liver cancer in Japan [25–27]. Notwithstanding that hepatitis B viral (HBV) infection is a major cause of HCC in Asian countries, for example Taiwan [28], Japan has a predominance of HCV-related HCC, and 70 and 16% of cases of HCC are ascribable to HCV and HBV infection, respectively [27–29]. Regional differences in HCV prevalence rate have been noted, namely higher rates in the western areas and lower rates in the eastern areas of Japan. Additionally, death due to hepatocellular carcinoma is high in west Japan [30]. Although Yusho patients are concentrated in the western part of Japan [11], there is no evidence that their carrier rate was higher than that in the background population. Potential misclassification bias may increase or reduce risk of malignant and non-malignant hepatic diseases. Thus, our results might have been biased as a result of misclassification of liver cancer and hepatic disease, which might in turn have lead to the overestimated liver cancer mortality.

Consistent with previous findings from our 40-year follow-up study [11], lung cancer mortality increased among males during the 50-year follow-up period. Additionally, a 30-year follow-up evaluation of the Yucheng cases and a meta-analysis of the Yusho and Yucheng cohorts found increased lung cancer mortality among males [12, 16]. A long-term follow-up study of workers with PCB exposure has shown that carcinomas of the respiratory system, and also of the subcategories of trachea, bronchi and lungs were substantially decreased in male employees, but increased in female employees [18]. The lung is a target organ of the carcinogenic action of dioxins in animals [31], and a recent laboratory-based study suggested that PCBs may have carcinogenic effects in human lung fibroblast cell line (HELF) cells at higher doses [32]. Although we have no data on smoking behaviors for each patient, the risks of smoking-related cancers, such as stomach, rectum, and pancreas, were not increased among Yusho patients. Furthermore, the possibility of a marked difference in cultural and educational features between Yusho patients and the general Japanese population is unlikely. Thus, we assume that smoking habits might not affect lung cancer mortality among Yusho patients.

Although recent case-control studies suggested positive associations between breast cancer and PCBs [33–35], we found no increase in breast or uterine cancer mortality. Our results accord with findings from previous Yusho and Yucheng cohort studies [11, 12, 16]. A recent prospective population-based Swedish cohort study also suggested that exposure to PCBs does not play a substantial role in the pathogenesis of cancers of the breast, endometrium or ovary [36]; in contrast, another population-based cohort study in the United States showed that estrogenic PCB congeners can adversely impact short- and long-term survival following a diagnosis of breast cancer [37]. Recent meta-analyses found that PCB exposure might increase the risk of breast cancer [38, 39]. Interaction between PCB exposure and genetic polymorphism in humans should be kept in mind when the adverse effects of PCBs are evaluated [38]; thus, more attention should be paid to PCB exposure in epidemiological studies for longer study periods when evaluating the association with breast cancer.

We observed no excess deaths for cancers of the stomach, rectum, or lymphatic and hematopoietic tissue in Yusho patients during the 50-year follow-up period. Additionally, although a recent study suggested the association of dioxin exposure with cutaneous malignant melanoma [40], we observed no cases of melanoma among Yusho patients. These results accord with those of our previous study of the Yusho cohort [11] and a meta-analysis of the Yusho and Yucheng cohort [12]. An update of a mortality assessment of employees exposed to PCBs also suggested that mortality from stomach cancer was within expectations [41]. Further, a recent hospital-based case-control study [42] and meta-analyses [43, 44] do not support a hypothesized association between exposure to PCBs and risk of cutaneous malignant melanoma. Nevertheless, a recent study also provided evidence suggesting a linkage between PCB exposure and the pathogenesis of chronic lymphocytic leukemia [45]. Further studies are needed to address these issues.

Regarding non-cancer-related diseases, Yusho patients showed no excess mortality for diabetes mellitus, hypertension, heart disease, or cerebrovascular disease. In contrast, Yucheng patients had elevated heart disease mortality. This discrepancy may be due to the fact that Japanese people have lower heart mortality than the Taiwanese population, since heart diseases have been increasing in Yucheng as a result of accelerated atherosclerosis [12, 46]. However, recent studies indicate that dietary exposure to PCBs is associated with cardiovascular disease, heart failure, myocardial infarction, stroke, hypertension, reduced cognitive function and diabetes [47–51]. However, on comparison with Yusho or Yucheng patients, who ate food with high levels of PCB contamination over a brief period, potential PCB exposure via the diet is long-term and low-level. Besides, increased mortality from systemic lupus erythematosus (SLE) was observed among Yucheng patients [16, 52], while this was not observed among Yusho patients. Although the exact mechanism remains unclear, this discrepancy in findings between Yusho and Yucheng may be related to the difference in protein oxidation between Yusho and Yucheng. Previous studies indicated that elevated levels of nitrotyrosine was observed in the serum of Yusho patients [53]. Nitrotyrosine is a marker of protein oxidation in sera and is associated with disease severity in SLE patients [54]. Further evaluation is needed to clarify whether PCB is associated with non-cancer-related diseases in Yusho patients.

Although verification of the vital status of the Yusho cohort was almost complete, and unlikely to have been confounded by major selection bias, there are several limitations in this study. First, bias might have occurred due to our inability to consider information about individual factors, such as socioeconomic and demographic factors, because of difficulties in collecting baseline data. In particular, we could not control for important confounding factors such as smoking behavior for lung cancer or viral hepatitis for liver cancer. Further, there may be also residual confounding due to migration and differences in place of residence. These potential biases may affect the interpretation of our results. Second, because of limited exposure data on the blood levels of PCBs and dioxins after the Yusho incident, our study could not identify any dose-response relationships between exposure and increased risk of mortality. Estimates of exposure at the individual level are required to conduct quantitative risk assessments. Moreover, many cancers which are known or presumed ascribable to environmental chemicals have extended latency periods, and it is important to improve estimates of exposure at the individual level by considering the time window of exposure [20]. Additional studies to estimate time-dependent dose-response associations may allow a more sophisticated characterization of mortality risk and a more valid risk assessment for policy and decision makers. Future studies should consider these important points.

## Conclusions

In summary, our study provides comprehensive data on Yusho patients with 50 years of follow-up, and confirms that the mortality risk ascribable to all cancer and lung cancer increased significantly among males compared with the general population. Furthermore, the SMRs for liver cancer increased significantly among females. Our findings suggest that carcinogenic risk to humans after exposure to PCBs and PCDFs remains higher among Yusho patients, and also the importance of care engagement and optimum management in controlling the burden of Yusho disease. There is no consistent knowledge about the effects of PCB and PCDF exposure on several types of cancer and non-cancer-related diseases, and further longer-term investigation is warranted.

## Data Availability

The data have been obtained through a restricted data use agreement with the Ministry of Health, Labour and Welfare, Japan, and are therefore not available for public dissemination.

## Abbreviations

PCBs: Polychlorinated biphenyls
PCDDs: Polychlorinated dibenzodioxins
PCDFs: Polychlorinated dibenzofurans
